# *Dorema kopetdaghense* Pimenov: A potent antifungal medicinal plant

**DOI:** 10.22034/cmm.2024.345183.1499

**Published:** 2024-05-07

**Authors:** Abolfazl Shakeri, Aliakbar Mashhadi Esmaeilabadi, Vahid Soheili, Javad Mottaghipisheh, Seyed Ahmad Emami, Javad Akhtari, Zahra Tayarani-Najaran

**Affiliations:** 1 Department of Pharmacognosy, School of Pharmacy, Mashhad University of Medical Sciences, Mashhad, Iran; 2 Department of Pharmaceutical Control, School of Pharmacy, Mashhad University of Medical Sciences, Mashhad, Iran; 3 Department of Aquatic Sciences and Assessment, Swedish University of Agricultural Sciences, SE, 75007, Uppsala, Sweden; 4 Department of Traditional Pharmacy, School of Pharmacy, Mashhad University of Medical Sciences, Mashhad, Iran; 5 Immunogenetics Research Center, School of Medicine, Mazandaran University of Medical Sciences, Sari, Iran; 6 Department of Pharmacodynamics and Toxicology, School of Pharmacy, Mashhad University of Medical Sciences, Mashhad, Iran

**Keywords:** Apiaceae, Antimicrobial, *Candida albicans*, Cytotoxicity

## Abstract

**Background and Purpose:**

*Dorema* species are well-known antifungal medicinal plants. *Dorema kopetdaghense* (Apiaceae family) is a rarely investigated plant endemic to Iran. The present study aimed to assess the antifungal, antibacterial, antioxidant, and cytotoxic activities of root extracts of different plants.

**Materials and Methods:**

The methanolic crude extract (MeOH) and its sub-fractions, including petroleum ether (PE), dichloromethane (DCM), ethyl acetate (EtOAc), and n-butanol (n-BuOH) were prepared.

**Results:**

Results from the antifungal and antibacterial activities of fractions indicated remarkable antifungal effects against *Candida albicans* with minimum inhibitory concentration and minimum bactericidal concentration values of 10 µg/mL; however, no cytotoxicity was observed in the case of selected cancer cells. Moreover, methanolic soluble fractions showed good antiradical effects evaluated *via* DPPH and *β*-carotene bleaching tests possessing half-maximal inhibitory concentration (IC_50_) of 20.11 and 41.32 µg/mL, respectively, though it was less effective than positive controls ascorbic acid (8.47 and 31.71 µg/mL, respectively) and butylated hydroxytoluene (IC_50_: 10.29 and 33.55 µg/mL, respectively).

**Conclusion:**

It can be concluded that strong antifungal and antioxidant activities without notable cytotoxicity, suggest the potential safety of the plant to be used as a natural antifungal remedy as well as a preservative in the food industry.

## Introduction

The *Dorema* genus belonging to the Apiaceae family comprises 12 species majorly growing in Iran, Caucasus, and Southern Asia (i.e. Afghanistan, Pakistan, and Baluchistan) [ 1 , 2 ]. Through a few phytochemical investigations of the genus, various flavonoids, coumarins, along with sesquiterpene derivatives have been identified as the main components, in that order [ 3 ]. *Dorema kopetdaghense* is a plant endemic to Iran and Turkmenistan. The ethnomedicinal applications of the oleogum resin have been documented in Persia since 4,000 years ago [ 4 - 6 ]. Until now, the sesquiterpenes kopetdaghins A–E, two phytosterols daucosterol and stigmasterol 3-*O*-glucoside, along with two coumarins have been identified as the phytoconstituents of *D. kopetdaghense* aerial and root parts [ 7 , 8 ]. In the present study, unavailable bioactivity information and the folk medicinal application of *D. kopetdaghense* were the rationale for the preliminary investigation of the antifungal, antibacterial, antioxidant, and antitumor potencies of its various extracts.

## Materials and Methods

### Plant Materials and Extract Preparation

*Dorema kopetdaghense* was collected at Dargaz (Kharasan-Razavi Province) in the Northeast of Iran in June 2018. The botanical identification was performed by Mr. Joharchi and a voucher specimen (13,220) was deposited in the Herbarium of the School of Pharmacy of Mashhad University of Medical Sciences in Mashhad, Iran. The root parts (50 g) were implemented *via* methanolic crude extract (MeOH, 3×250 mL) in the extraction process using the maceration method. After filtration, the solvent was removed using Rotavapor (Heidolph, Germany) under reduced pressure at 40 °C. Application of different polar solvents using a separating funnel led to the acquisition of petroleum ether (PE), dichloromethane (DCM), ethyl acetate (EtOAc), and n-butanol (n-BuOH) soluble fractions. The partitions were concentrated and then stored at 4 °C for the experiments.

### Antibacterial and antifungal activities

The broth dilution method introduced by a standard method was applied with slight modification to ascertain the antimicrobial activities of the plant samples by determination of the minimum inhibitory concentration (MIC) and minimum bactericidal concentration (MBC) values [ 9 , 10 ]. The microorganisms, including Gram-positive *Bacillus cereus* (PTCC: 1247), Gram-negative *Pseudomonas aeruginosa* (PTCC: 1707), *Salmonella typhi* (PTCC: 1609), *Escherichia coli* (PTCC: 0157) bacteria, and the fungus *Candida albicans* (PTCC: 5027) were provided by the Microbiology Laboratory of Mashhad University of Medical Sciences. The 96-well microtiter plates were used to measure the MIC values of the samples for each bacteria culture. The wells in columns 1 to 8 were allocated to the extracts, individually, where the treatments were performed as follows: 180 µL of 640 µg/mL in the first column, 180 µL of 320 µg/mL in the second column, and this continued until the eighth column contained 5 µg/mL of each extract. Thereafter, 20 µL microbial suspension (10^6^ CFU/mL) was added to each well. The rows A–C and D–F each were assigned to one strain, whilst genthamycine (5 µg/mL) and ketoconazole (250 µg/mL) as the positive controls for the experimented bacterium and fungus, respectively, were added to row G in addition to 0.1 mL microbial suspension. To sterilize the medium culture, 200 µL sterile Mueller Hinton broth was added to the well H1, while 180 µL of medium culture and 20 µL microbial suspension were added to the H11 and H12 wells to provide the negative control. The plates containing bacteria and fungus were incubated at 37 °C for 24 and 48 h, respectively. Afterward, 20 µL tetrazolium (2,3,5-triphenyl-tetrazolium chloride) in an aqueous solution (5 mg/mL) was added to each plate and then incubated at 37 °C for 3 h. The MIC values were subsequently analyzed and presented as µg/mL. In case no color change was detected in the wells, 10 µL was taken and incubated for 18–20 min on the soyabean casein digest agar culture. The MBC values (µg/mL) were measured where no growth was observed.

### Antiradical activity

### DPPH assay

The DPPH assay was used to assess free radical scavenging potencies of the plant extracts [ 11 ]. In brief, different microdilution series of the extracts (10–100 µg/mL) were dissolved in MeOH on 96-well microtiter plates. Thereafter, 1 mL of DPPH solution (0.2 mM) was individually added to 2 mL of each sample. After storing them at room temperature in dark conditions for 30 min, their absorbance was read at 517 nM by UV-Vis spectrophotometer (Cecil™, the exact model, England). butylated hydroxytoluene (BHT) and ascorbic acid (AA) (Merck, Germany) as positive and methanol (MeOH, high-performance liquid chromatography grade) as blank controls were exploited. 

### β-carotene bleaching assay

The antioxidant properties of the extracts were further examined by *β*-carotene bleaching (BCB) assay [ 12 ]. Briefly, 1 mL of *β*-carotene solution (0.2 mg/mL) in chloroform was added to the mixture of linoleic acid (20 mg) and Tween 40 (100 mg) in a boiling flask. Chloroform was evaporated under a vacuum at 50 °C, 50 mL of distilled water was added, and then the solution was sonicated for 1 min to eventually obtain emulsion A. The emulsion B was prepared by sonication (1 min) of the mixture of linoleic acid (20 mg), Tween 40 (200 mg), and distilled water (50 mL). Consequently, the ethanolic stock solutions of samples (200 µL) in different concentrations were mixed with emulsion A (5 mL) in a test tube. A negative control was also attained by mixing 200 µL ethanol and 5 mL emulsion A. The UV-Vis spectrophotometer was set to zero using a solution containing ethanol (200 µL) and 5 mL of emulsion B. Absorbance of blank (negative) control was immediately read at 470 nm (t: 0 min). The samples were incubated at 50 °C for 120 min, then the absorbanc es were recorded. 

### Cytotoxicity assessments

The cytotoxic activities of the different plant extracts against three human cancer cell lines MCF-7 (breast adenocarcinoma), A549 (lung carcinoma), and PC3 (prostate) were elaborated utilizing AlamarBlue™ assay. The cells were provided by the Pasteur Institute of Iran. Following this fluorometric method, resazurin (7-hydroxy-3H-phenoxazin-3-1-10-oxide) is reduced to resorufin by mitochondrial enzymes of metabolically active cells while transforming pale bluish color of resazurin to highly fluorescent reddish color of resorufin is used as cell viability indicator [ 13 ]. As previously described [ 14 ], a 96-well flat-bottomed microtiter plate (1×10^4^ cells for each well) was used for cell culturing. The extracts with concentrations of 6.25, 12.5, 25, 50, and 100 μg/mL were individually inserted into each well, then incubated for 48 h at 37 °C and 5% CO_2_ (v/v), Subsequently, AlamarBlue™ solution (10 μL) was added. After 4 h of incubation, the absorbances were recorded at 600 nm by exploiting a microplate spectrophotometer reader (BioTek, Epoch™, USA). The cytotoxic activities were expressed as half-maximal inhibitory concentration (IC_50_) values assessed by Graph Pad software (Graph Pad Prism V.6).

## Results

### Antioxidant properties

**Figure 1:**
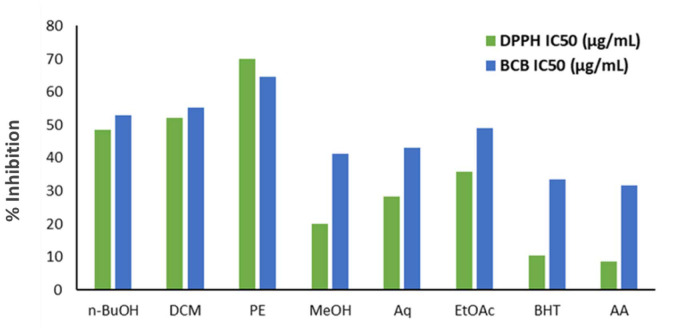
Antioxidant activities of different *Dorema kopetdaghense* extracts evaluated by DPPH and BCB assays. *n*-BuOH: *n*-butanol, DCM: dichloromethane, PE: petroleum ether, MeOH: methanol, Aq: aqueous, EtOAc: ethyl acetate, BHT: butylated hydroxytoluene, AA: ascorbic acid.

According to the DPPH assay, the methanolic extract exhibited the highest free radicals inhibition possessing the lowest IC_50_ value (Figure 1), although BHT and ascorbic acid were more potent as positive controls. The antiradical properties of the extracts were analyzed as follows: AA (IC_50_: 8.47 µg/mL) > BHT (IC_50_: 10.29 µg/mL) > MeOH (IC_50_: 20.11 µg/mL) > Aq (IC_50_: 28.37 µg/mL) > EtOAc (IC_50_: 35.92 µg/mL) > *n*-BuOH (IC_50_: 48.61 µg/mL) > DCM (IC_50_: 52.10 µg/mL) > PE (IC_50_: 70.01 µg/mL).

The BCB results (Figure 1) affirmed the DPPH assay. Accordingly, among the extracts of the plant, methanolic fraction demonstrated the highest potency, in comparison with positive controls which were BHT and ascorbic acid. The effects were determined based on the following order: AA (IC_50_: 31.71 µg/mL) > BHT (IC_50_: 33.55 µg/mL) > MeOH (IC_50_: 41.32 µg/mL) > Aq (IC_50_: 43.10 µg/mL) > EtOAc (IC_50_: 49.06 µg/mL) > *n*-BuOH (IC_50_: 53.02 µg/mL) > DCM (IC_50_: 55.18 µg/mL) > PE (IC_50_: 64.61 µg/mL).

### Antimicrobial activities

The extracts indicated antimicrobial effects against the tested bacteria and fungus. Notably, all the plant fractions significantly possessed a growth inhibition effect on *C. albicans* with MIC and MBC values of 10 µg/mL (Table 1). 

**Table 1:** Antimicrobial potencies of different extracts against the selected microorganisms; MIC and MBC values are depicted as µg/mL.

PTCC Strains	Extracts									
	MeOH		PE		DCM		EtOAc		*n*-BuOH	
	MIC	MBC/MFC	MIC	MBC/MFC	MIC	MBC/MFC	MIC	MBC/MFC	MIC	MBC/MFC
*Bacillus cereus* (1247)	80	80	10	10	10	10	80	80	40	40
*Pseudomonas aeruginosa* (1707)	n.a*	n.a	n.a	n.a	n.a	n.a	n.a	n.a	n.a	n.a
*Salmonella typhi* (1609)	n.a	n.a	160	360	40	80	160	320	n.a	n.a
*Escherichia coli* (0157)	n.a	n.a	n.a	n.a	n.a	n.a	n.a	n.a	n.a	n.a
*Candida albicans* (5027)	10	10	10	10	10	10	10	10	10	10

* PTCC: Persian type culture collection, Not active (>500 µg/mL), *n*-BuOH: *n*-butanol, DCM: Dichloromethane, PE: Petroleum ether, MeOH: Methanol, Aq: Aqueous, EtOAc: Ethyl acetate, MIC: Minimum inhibitory concentration, MBC: Minimum bactericidal concentration, MFC: Minimum fungicidal concentration

### Cytotoxic activities

The plant’s soluble fractions at the experimented concentrations (3.125-100 µg/mL) revealed no cytotoxicity against MCF-7, A549, and PC3 cell lines, nevertheless, IC_50_ values for doxorubicin (the positive control) were recorded as 0.25, 0.49, and 0.99 µg/mL, respectively.

## Discussion

According to the results, all the plant extracts illustrated potent anticandidal activities indicating MIC and MBC values of 10 µg/mL. *Candida albicans* is the most common *Candida* species, well-known as a human pathogen responsible for many infections, including vaginal candidiasis, onychomycosis, cutaneous candidiasis, and chronic mucocutaneous candidiasis [ 15 ]. Suppression of this aggressive fungus has been studied by many investigations, particularly by natural product chemists. Terpenoids, including sesquiterpenes as the major compounds formerly identified from the studied plant, exhibited compelling effects against *C. albicans* [ 16 , 17 ]. Moreover, triterpenoid glycosides inhibited the growth of some human pathogens, such as *Candida* and *Cryptococcus* species [ 18 ]. Therefore, this high activity was most probably caused by terpenoid contents which highlighted further antifungal evaluations of its phytoconstituents against diverse fungal species. It is worth mentioning that no cytotoxicity was observed in the case of three cancer cells up to 100 µg/mL. Results of the present study were in agreement with those of a previous study which indicated that the aqueous extracts of another species of *Dorema* (*Dorema ammoniacum*) exhibited no toxicity against human red blood cells up to a concentration of 300 mg/mL [ 19 ]. Based on the current outcomes, the polar plant extracts revealed a higher antioxidant effect, specifically, the methanolic fraction; this can be correlated to the phenolic contents, where these compounds, as the major natural radical scavengers, are medium to high polar, accordingly richer in polar fractions [ 20 ].

## Conclusion

The present research analyzed the antifungal, antibacterial, antioxidant, and antitumor properties of different extracts obtained from the root part of *D. kopetdaghense*, for the first time. Conclusively significant antifungal effects of various extracts against *C. albicans* and the antioxidant potential of methanolic extract were observed. The present preliminary findings introduced a broad promising approach for futuristic phytochemical and biological investigations of *D. kopetdaghense*, where the bioassay-guided fractionation, isolation, and characterization of the plant extracts can lead to the discovery of the responsible antifungal compounds. In addition, potent antifungal and antioxidant activities without notable cytotoxicity, suggest the potential safety of the plant to be used as a natural antifungal remedy as well as a preservative in the food industry.
